# A 454 Survey Reveals the Community Composition and Core Microbiome of the Common Bed Bug (*Cimex lectularius*) across an Urban Landscape

**DOI:** 10.1371/journal.pone.0061465

**Published:** 2013-04-09

**Authors:** Matthew Meriweather, Sara Matthews, Rita Rio, Regina S. Baucom

**Affiliations:** 1 Department of Biological Sciences, University of Cincinnati, Cincinnati, Ohio, United States of America; 2 Department of Biology, West Virginia University, Morgantown, West Virginia, United States of America; University of Utah, United States of America

## Abstract

Elucidating the spatial dynamic and core constituents of the microbial communities found in association with arthropod hosts is of crucial importance for insects that may vector human or agricultural pathogens. The hematophagous *Cimex lectularius* (Hemiptera: Cimicidae), known as the human bed bug, has made a recent resurgence in North America, as well as worldwide, potentially owing to increased travel, climate change and resistance to insecticides. A comprehensive survey of the bed bug microbiome has not been performed to date, nor has an assessment of the spatial dynamics of its microbiome. Here we present a survey of internal and external bed bug microbial communities by amplifying the V4–V6 hypervariable region of the 16S rDNA gene region followed by 454 Titanium sequencing using 31 individuals from eight distinct collection locations obtained from residences in Cincinnati, OH. Across all samples, 97% of the microbial community is made up of two dominant OTUs, previously identified as the α-proteobacterium *Wolbachia* and an unnamed γ-proteobacterium from the Enterobacteriaceae. Microbial communities varied among host locations for measures of community diversity and exhibited structure according to collection location. This broad survey represents the most in-depth assessment, to date, of the microbes that associate with bed bugs.

## Introduction

The interactions between microbes and their insect hosts can range from obligate mutualism to parasitic and ultimately detrimental to host fitness [1], [2], [3]. The microbes that insects carry can also negatively impact other organisms following vector transmission. Characterizing the microbial community of insect hosts is thus an important primary step for elucidating the nature of host/microbe interactions and the potential for the insect vectoring of important pathogens. Assessments of the core microbiome-defined as members of the microbial community that are common to two or more assemblages of a particular habitat type [4], [5]- are often performed to identify key host/microbe associations. The microbes that appear in all hosts are thought to fulfill a functional niche within the community [6] and as such may provide valuable information on the ‘normal’ state of the community as well as on what members might be targeted for manipulation (e.g., in a scenario of insect biocontrol).

The microbiome of an insect host could vary among host populations, and such natural variation would represent one aspect of an ecological deviation from the core microbiome [6]. Although assessments of the microbiome of various arthropods is an area of very active research [7], [8], [9], [10], [11], [12], [13], [14], [15], the potential for natural, spatial variation in the microbiome has been elucidated in only a handful of insect species [8], [10], [16]- perhaps most thoroughly in the model species *Drosophila* [9], [17]. Interrogating spatial variation in the core microbiome of a range of insects, and specifically arthropods that may vector disease is of interest for three main reasons: first, the maintenance of a cohort of microbial species across many insect populations may provide information about important components of host physiology; second, the potential for interaction between endosymbiont species may be elucidated by wide-ranging surveys of microbial taxon abundance and frequency of co-occurrence, and finally, surveys of the spatial variation in microbiomes may identify populations in regions of importance to human and/or agricultural health that may then be targeted for remediation.

Members of the Cimicidae family (bed bugs) are obligate ectoparasites obtaining blood meals from birds, bats and humans (Usinger 1966). The medically significant *Cimex lectularius*, the human bed bug, has made a now infamous resurgence in North America as well as worldwide [18], [19], [20], [21]. Bed bug infestations, while not restricted to social or economic groups, can disproportionately impact the economically disadvantaged since the efficacy of less expensive methods of control, such as pesticide application, is low owing to widespread pyrethroid resistance [22], [23]. While there is currently no evidence that bed bugs vector disease, human pathogens have been identified on the surface of the insects and in their excrement [24]. Further, the presence of this hematophagous insect in dwellings can lead to skin reactions, anaphylaxis, anxiety and psychological problems in humans [24], [25], [26], and thus bed bugs are of broad and increasing concern from the standpoint of human health [27].

Endosymbiont microbial species in *C. lectularius* were first identified by microscopy in 1921 [28], and recent 16S rRNA surveys have identified two main endosymbionts of the bed bug to be *Wolbachia* [29], [30], [31] and an unnamed, γ-proteobacterium closely related to an endosymbiont species from the leafhopper *Euscelidius variegatus* (termed BEV-like symbiont by[29]). A high prevalence of *Wolbachia* infections has been found in North American and Japanese populations of *C. lectularius* (between 83–100%, [32]), and infection rates of the γ-proteobacterium are reported to vary from 0–100% among locations in Japan [33]. Further, 16S rRNA sequencing has identified the strain of *Wolbachia* found in *C. lectularius* to belong to the F supergroup [30], [32], [33]. Unlike the B supergroup of *Wolbachia* that parasitizes many arthropods, the F supergroup, described within both arthropods and nematodes, is not known to harbor any reproductive parasites [33]. Recent work has shown that *Wolbachia* is a nutritional mutualist of *C. lectularius* and provides the species with B vitamins essential for growth and reproduction [33]. For this reason, *Wolbachia* has been proposed as an obvious target for the biocontrol of this insect pest [33]. However, before such an endeavor can be expected to succeed, more information regarding the natural microbial community of *C. lectularius* is needed. A broad assessment and characterization of the core microbiome of bed bugs collected from an urban area is thus highly warranted.

**Figure 1:**
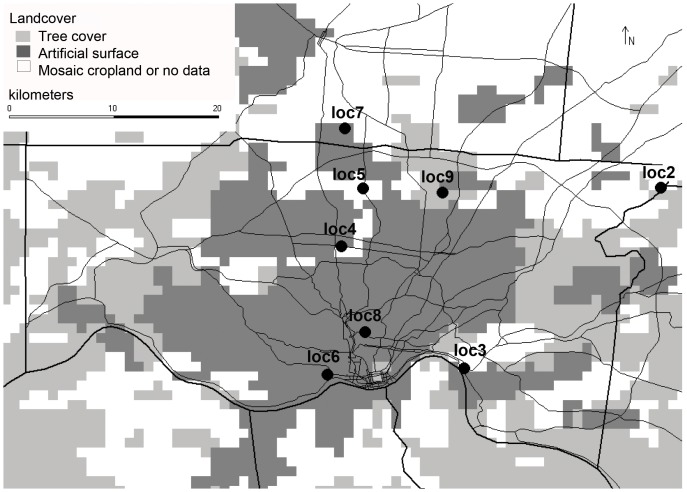
Map of *Cimex lectularius* collection locations from the greater Cincinnati metropolitan area in Cincinnati, OH, USA.

Here, we surveyed the microbiome of bed bugs obtained from residences located in metropolitan Cincinnati, OH (Fig 1), which is currently listed as the second most bed bug infested US city [34], [35]. To make our preliminary assessment as geographically comprehensive as possible, we surveyed between 2 and 5 *C. lectularius* individuals from each of eight geographically separated collection locations. We used culture-independent 16S rDNA PCR amplification followed by 454 Titanium sequencing to characterize the microbial community associated with each *C. lectularius* individual. We performed this survey to broadly characterize the core microbiome of naturally collected bed bug samples and to assess the following: Are there differences in the bed bug microbial community diversity and/or membership among collection locations? Are the two dominant and previously described endosymbionts (*Wolbachia* and an unnamed γ-proteobacterium) present across all collection locations? Is there any evidence for pathogenic microbial groups within collection locations of *C. lectularius* sampled from local residences?

## Results

### Data summary

Following DNA extraction from *C. lectularius* individuals, the hypervariable V4–V6 region of the bacteria-specific 16S rDNA gene was PCR amplified in triplicate for each individual. Libraries from the same individual were then combined, cleaned, normalized, and sequenced using 454 Titanium chemistry.

From the initial 69,854 sequences, 11,147 were removed for being <200 bp or low quality, 3,927 were identified as chimeric, and 666 were identified as either mitochondrial or chloroplast. After filtering the sequence reads for quality scores, sequencing errors and chimeras, our dataset consisted of 54,114 sequences distributed across 31 *C. lectularius* individuals (also referred to as libraries)(Table S1) with an average read length of 284 bases. The 31 libraries varied in size from 74 to 4092 sequences, and averaged 1745.6±171.2 sequences. Most libraries (25) contained at least 1000 sequences. Clustering the data with mothur [36] at the 3% level of sequence divergence returned 359 OTUs across all libraries. Two OTUs overwhelmingly dominated the sequence data, with one OTU containing 34,354 sequences and the other prominent OTU containing 18,085 sequences, which cumulatively constitute approximately 97% of all sequences. On average, OTUs contained 150 sequences, but many OTUs (239) were represented by 1 or 2 sequences. Twenty-nine OTUs were represented by 10 or more sequences. The taxonomic membership of collection locations (described below) was assessed using reads from all libraries and reported in Table 1, whereas analyses of community structure and diversity were performed following a random sampling of sequences from the libraries that contained at least 1000 sequences (see Methods; Table S1).

**Table 1:** The relative abundance as the proportion of 16S rRNA sequences that mapped to the 10 most abundant OTUs shared across all or most locations.

OTU ID Number	Phylum/Class/Suborder or Family	Genus	Abundance	Number of libraries OTU present	Number of locations OTU present
	**Actinobacteria**				
	Actinobacteria				
29	Frankineae	Unclassified	0.23	18	7
4	Propionibacterineae	Unclassified	0.11	21	8
31	Streptomycineae	Unclassified	0.07	17	7
	**Proteobacteria**				
	Alphaproteobacteria				
1	Anaplasmataceae	*Wolbachia*	63.48	31	8
26	Rhizobiaceae	*Shinella* genera *(incertae sedis)*	0.22	16	8
23	Sphingomonadaceae	*Sphingomonas*	0.13	22	8
15	Methylobacteriaceae	*Methylobacterium*	0.10	25	8
	Gammaproteobacteria				
2	Enterobacteriaceae	Unclassified	33.42	31	8
85	Pseudomonadaceae	*Pseudomonas*	0.16	16	8
11	Enterobacteriaceae	Unclassified	0.15	20	8

The number of individual *C. lectularius* (*i.e.* libraries) that exhibit each OTU is presented along with the number of locations with the OTU.

### Bacterial taxa associated with wild *Cimex* populations

Approximately 97% of all sequences within our dataset were assigned to two families within the Proteobacteria by the RDP classifier: Anaplasmataceae (63.48%) and the Enterobacteriaceae (33.57%). Two genera-*Wolbachia* and an unclassified genus from the Enterobacteriaceae (hereafter ‘unnamed γ-proteobacterium’)- comprised the majority of these families, at 63.48% and 33.42%, respectively (Table 1). A blastn analysis of a representative sequence from the unnamed γ-proteobacterium shows high similarity (99%, e-value = 3e^−139^) to a previously identified maternally transmitted γ-proteobacteria endosymbiont from *C. lectularius* that was termed ‘BEV-like’ endosymbiont [29], [33]. The remaining OTUs from our dataset comprise roughly 3.10% of the sequences (Table S2). All 31 individual *C. lectularius* libraries contained the dominant *Wolbachia* and the unnamed γ-proteobacterium; all individuals from eight collection locations had a shared OTU from each of the following genera at very low frequency: *Shinella*, *Sphingomonas*, *Methylobacterium*, *Pseudomonas*, a second unclassified genus from the Enterobacteriaceae (OTU 11), *Acinetobacter*, and *Cloacibacterium*, as well as a shared OTU from the suborder Propionibacterineae (Table 1 and Table S2). Thus, a core microbiome in wild-collected populations of *Cimex* could be expected to contain *Wolbachia* and the γ-proteobacterium and possibly OTUs from the low-frequency groups listed above. We screened our data for putative pathogens that were previously found to associate with *Cimex* individuals [24], and while the length of the 16S fragment that we analyzed precludes the identification of bacteria at the species level, we detected five bacterial OTUs from the same genera (*Bacillus*, *Coxiella*, *Staphylococcus*, and *Streptococcus*) as a potentially human-pathogenic bacterial species (Table 2).

**Table 2:** Genera detected in the wild populations of *C. lectularius* previously detected in either wild or laboratory strains of bed bugs (^1^from Delaunay et al 2011).

Genus detected in this study	Number of OTUs identified	Potential pathogen previously detected in *C. lectularius* or *C. hemipterus* ^1^
*Bacillus*	2	*Bacillus anthracis*
*Coxiella*	2	*Coxiella burnetii* (Q fever)
*Staphylococcus*	1	*Staphylococcus aureus*
*Streptococcus*	2	*Streptococcus pneumonia*
*Wolbachia*	3	*Wolbachia* spp

### Community structure of the *Cimex* microbial associates

**Figure 2:**
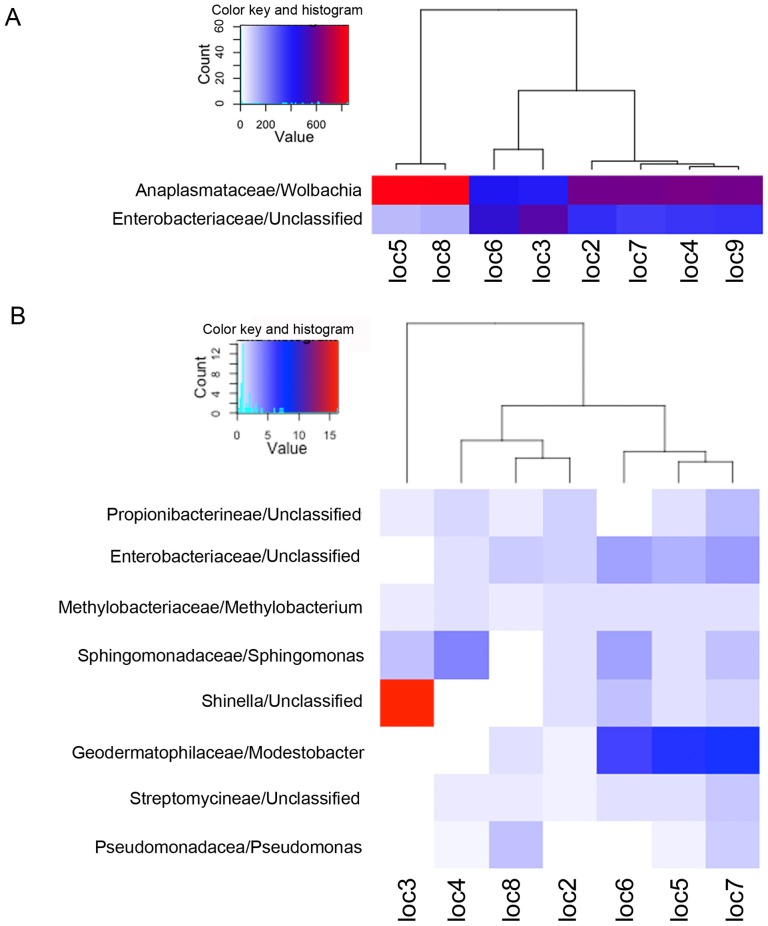
Composition and abundance of the *C. lectularius* microbiome within eight wild populations collected from Cincinnati, OH, USA. Each individual *C. lectularius* library was randomly subsampled to 1000 sequences, and the abundance of each OTU was averaged according to collection location. Note that location 6 is represented by a single individual. A. Relative abundance of the two most abundant bacterial families and genera within the locations. B. Relative abundance of the next 8 most abundant bacterial families and genera. Note the difference in scale in the respective keys for panels A and B.

While the core microbiome of *Cimex lectularius* individuals collected from urban Cincinnati is dominated by two OTUs from Proteobacteria (*Wolbachia* and the unnamed γ-proteobacterium), their relative abundances differed among collection locations (Fig 2A). *Wolbachia* was found to be highly abundant (84–85%) in locations 5 and 8, respectively, whereas the unnamed γ-proteobacterium was found at only 11–13% in these locations. In contrast, locations 6 and 3 exhibited slightly more of the unnamed γ-proteobacterium (56 and 49%) than *Wolbachia* (40 and 43%) (Fig 2A). Locations 2, 4, 7 and 9 showed similar relative abundances of these two genera on average (∼60% *Wolbachia* and ∼34% unnamed γ-proteobacterium). It is important to note that the relative abundance of *Wolbachia* and the γ-proteobacterium among collection locations reported here could be influenced by copy number variation of the 16S rDNA gene, either within or among populations. Although the 16S rDNA gene of *Wolbachia* is reported to be single copy from annotated genomes within the A, B and D supergroups [37], [38], [39] there is no record of copy number in the F supergroup of *Wolbachia*, nor is there a record for 16S rRNA copy number in the γ-proteobacterium.

The next 8 most abundant OTUs were detected in almost all locations but at very low frequency (Table 1). An OTU classified as *Shinella*, from the Rhizobiales order, was found in location 3 at 1.63% and yet at only 0.2% in location 6 and at lower frequency in all other locations (Fig 2B). An OTU from the Frankineae suborder was present at relatively similar frequency among locations 5, 6, and 7 (∼0.68%). These same locations also exhibited a relatively similar frequency as an OTU that was another member of the Enterobacteriaceae. The majority of our bootstrap values from the RDP classifier provided 100% support, especially for the high-frequency OTUs, with the taxonomic assignment of a few low-frequency OTUs between 80–90% (see Table S2).

**Figure 3:**
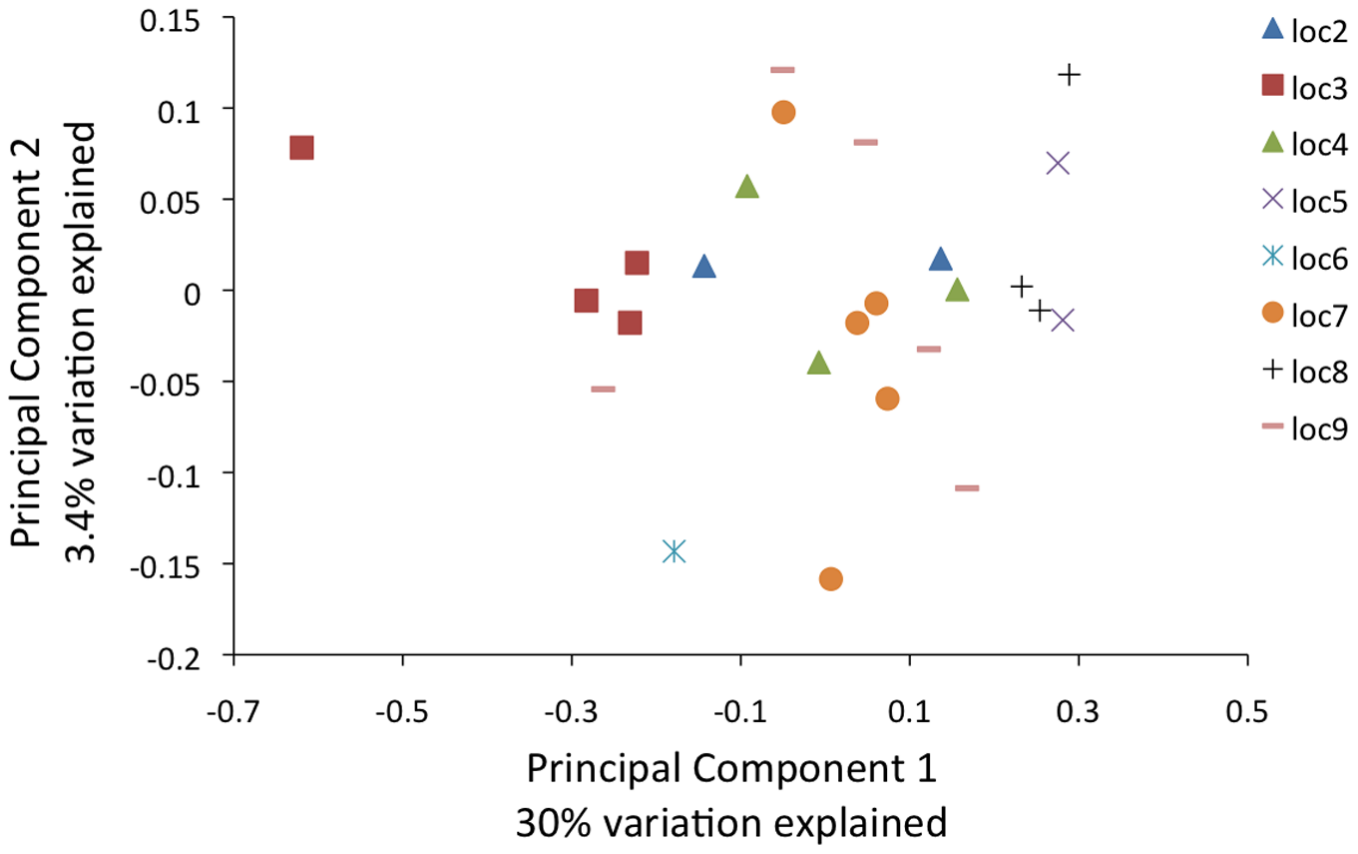
Principle component analysis of the *Cimex* microbiome. The Yue and Clayton measure of dissimilarity between the structures of the microbiome communities (θ-YC) was estimated and visualized using the dist.shared and pcoa commands of mothur [36].

To determine if the patterns of abundance represent significant microbial community structuring across collection locations, we estimated the Yue and Clayton similarity coefficient (θ)[40] among the individual *Cimex* microbiomes. The Yue and Clayton θ index is a similarity index that includes species proportions of both the shared and non-shared species in each population. The index ranges from 0 to 1, with 1 = complete similarity and 0 = complete dissimilarity. We then visualized the resulting distance matrices using a principle components analysis (PCoA) and performed an analysis of molecular variance (AMOVA) to determine if the spatial separation in the PCoA plot was statistically significant, *i.e*., individuals of the same location shared similar communities and at similar abundances. The PCoA plot (Fig 3; Movie S1) of this metric of community similarity shows evidence of structure in that individual microbiomes from the same location tended to cluster with one another; further, the AMOVA uncovered evidence that the variation among location spatial structure was greater than variation between individuals collected from the same location (F_7_ = 11.25, P = 0.008).

### Patterns of microbial diversity among the *Cimex* microbiomes

**Figure 4:**
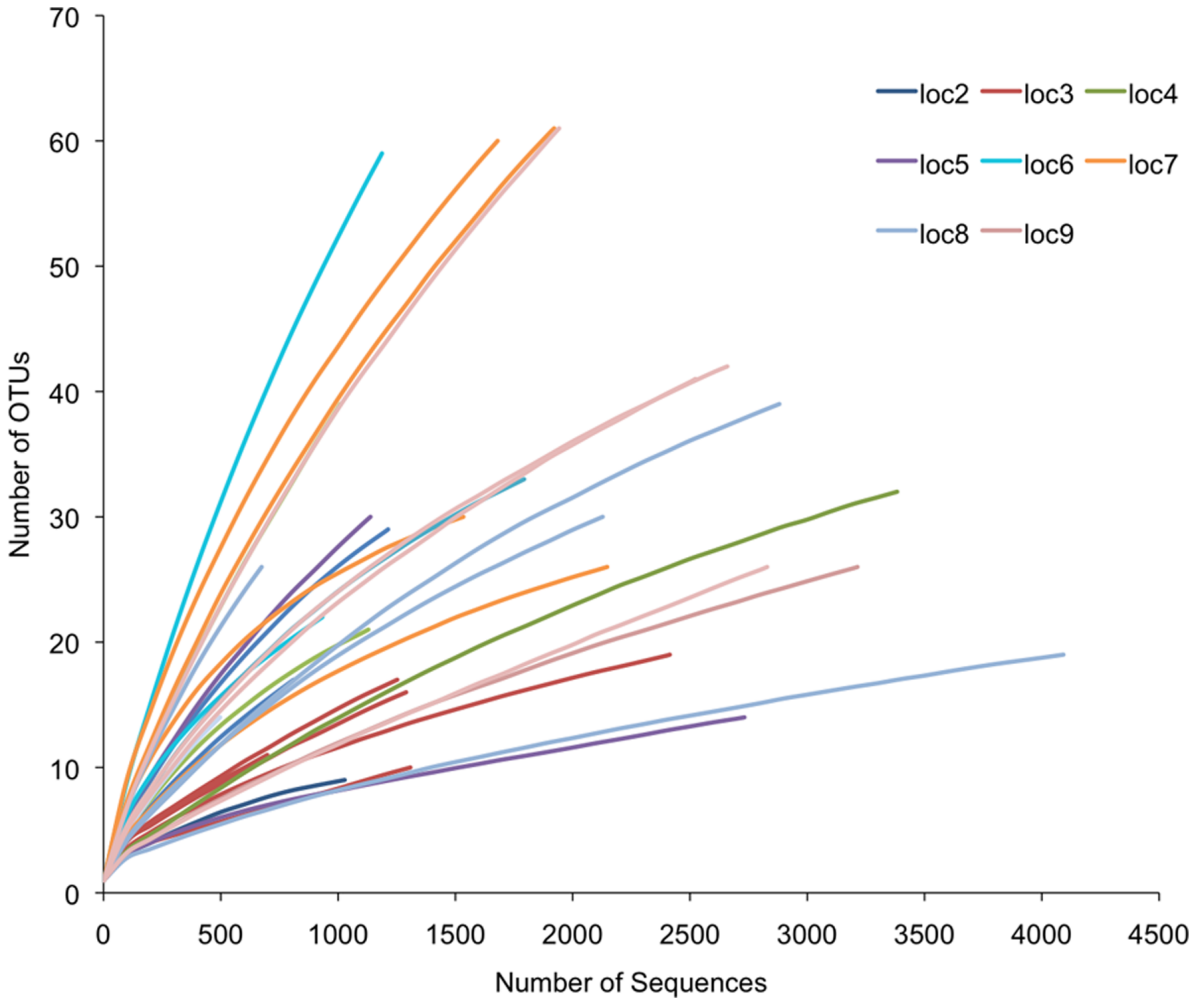
Rarefaction analysis of observed richness of the microbiome of *C. lectularius* individuals. All calculations were performed with mothur [36]. OTUs were defined at the 3% level of sequence divergence. Individuals collected from the same location are represented by the same color.

On average, 22.56±2.48 (SE) OTUs were uncovered across the locations, with four libraries either approaching or exhibiting approximately 60 OTUs (Fig 4). On the lower end, three libraries appeared to asymptote between 10–15 OTUs. Rarefaction analysis shows that individual *Cimex* microbiomes vary in richness (Fig 4), and that the libraries were sampled at different depths. Those that were not sampled to completion could harbor rare and potentially important bacterial taxa. Coverage of the *Cimex* microbiomes averaged at 98.5%, suggesting that, on average, 1.5 unique OTUs would be expected for every additional 100 sequences.

**Figure 5:**
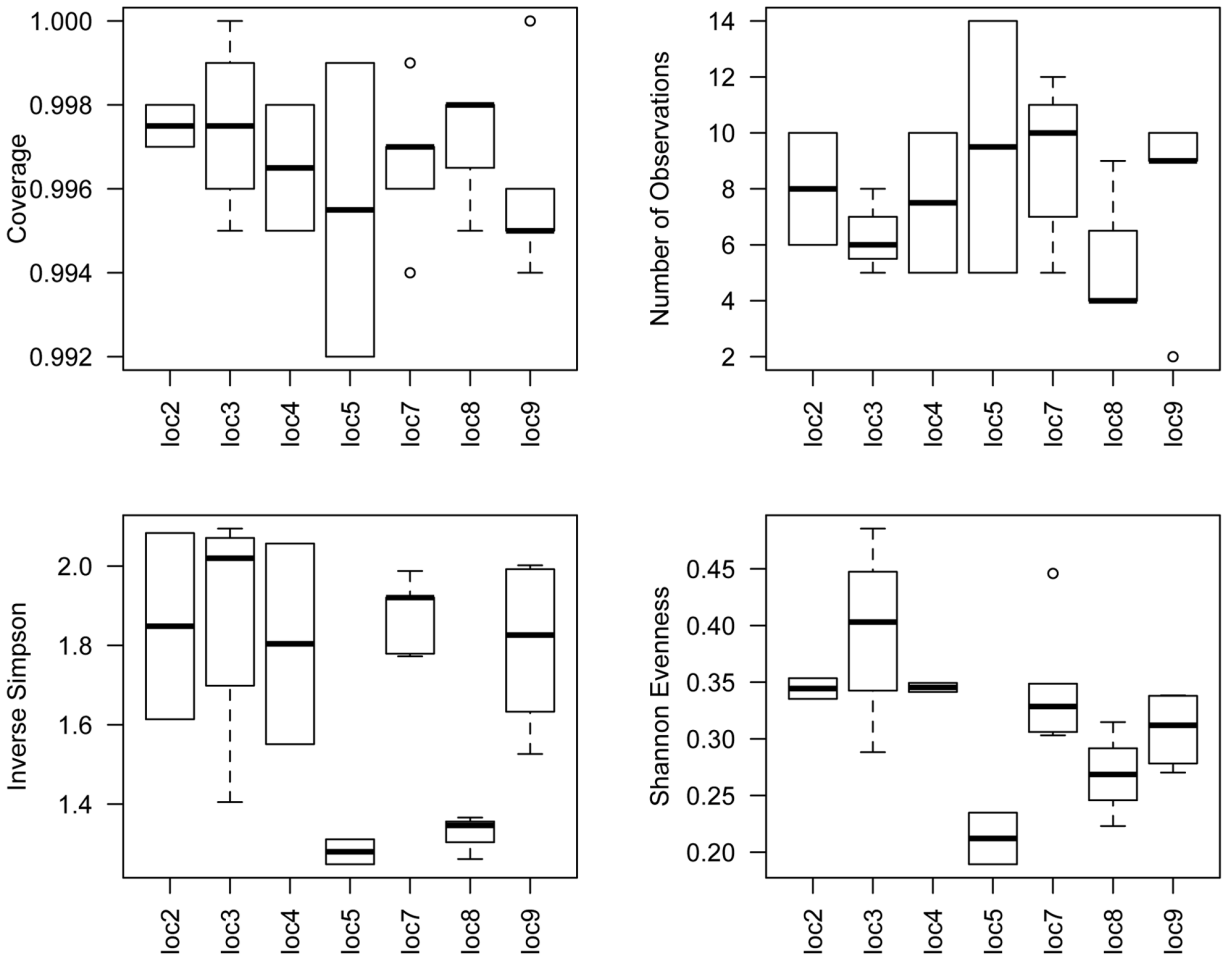
Boxplots of the diversity and evenness of the bacterial communities found in association with *C. lectularius* individuals, averaged according to collection location. Individual libraries were randomly subsampled to 1000 sequences each prior to estimating diversity parameters. Calculations were performed with mothur [36] at the 3% level of sequence divergence. The number of individuals within each population is presented in Table S1.

Indices of diversity and evenness varied significantly among locations (Fig 5; Tables S3A&B, Table S4), and this variation appeared to be driven largely by the relative low diversity and evenness of locations 5 and 8. These two locations were dominated by *Wolbachia* relative to the unnamed γ-proteobacterium, and exhibited an average of 18.5 (location 5) and 14.3 (location 8) OTUs. Rarefaction analysis of the Inverse Simpson index shows that unequal sampling among *Cimex* libraries did not excessively influence this estimate of diversity (Figure S1).

## Discussion

### The core *Cimex* microbiome

We found two dominant OTUs to make up 97% of the *C. lectularius* microbiome. The RDP classifier assigned these OTUs to the α-proteobacteria *Wolbachia*, an obligate endosymbiont, and an unnamed BEV-like γ-proteobacterium, closely related to a symbiont of the leafhopper *Euscelidius variegatus*. Both taxa have been described from previous surveys of *C. lectularius-*they are transovarially transmitted, persist in bacteriocytes (both taxa) [29], [33], [41] and/or are found in many cell types of the bed bug (unnamed γ-proteobacterium [33]). *Wolbachia* comprised the largest fraction of our microbiome survey (∼63%) and was present in all locations and individuals. Broader geographical assessments have previously indicated that *Wolbachia* infections are present in ∼95% of surveyed *C. lectularius* populations [32], and earlier experimental work suggested a role for this symbiont in *C. lectularius* fertility [42], [43], [44]. More recently, it has been shown that the presence of *Wolbachia* in bed bugs provides a fertility benefit through the production of B vitamins [33] supporting the idea that this taxon is an obligate nutritional mutualist of the bed bug. Thus, the high frequency of *Wolbachia* uncovered in this study is relatively unsurprising, but provides evidence that our short-read 16S rDNA assessment is largely congruent with expectations.

While different sub-types of *Wolbachia* are known to inhabit many arthropods and can range in association with their hosts along a parasitic to mutualistic continuum [45], [46], [47], there is comparatively less known about the lifestyle of the second most common OTU in our dataset, the unnamed γ-proteobacterium. Phylogenetic assessment of this taxon found it to exhibit greater than 99% similarity to BEV [29], a facultative endosymbiont reported from the leafhopper *Euscelidius variegatus* [48] and closely related to the plant pathogenic genera *Dickeya*, *Pectobacterium*, and *Erwinia* [49]. Close phylogenetic relationships between endosymbionts found in plant-feeding and blood-feeding insect hosts have been uncovered in other bacterial clades-*Rickettsia*, *Sodalis* and *Arsenophonus* [50], [51], [52]- and may represent putative examples of horizontal transfer, potentially due to their ability to persist outside of hosts [49]. While the phenotypic effects of this γ-proteobacteria on *C. lectularius* are largely unknown, there are accounts of pen-strep treatment (which does not affect *Wolbachia*) leading to reductions in egg production in *C. lectularius*, suggesting that the removal of the γ-proteobacteria can lead to reductions in fertility [41] and thus it may prove to be a nutritional mutualist as well.

### Identification of putative human pathogens in *Cimex*

One of the goals of this survey was to determine if human pathogenic bacteria could be detected within *C. lectularius* collected in a dense urban setting. Because *C. lectularius* colonies infest walls and other cracks and crevices of structures, live in high density in their excrement, and shuttle around dust, the environmental microbes (*i.e.* those that can be found on the exoskeleton) were of interest as were potential pathogenic endosymbionts. Although bed bugs have not been definitively shown to transmit disease, 45 human pathogens with the potential for transmission through bed bug vectors, most likely mechanical in contrast to true vector, have been previously identified within or on the insects and/or their excrement [24]. Furthermore, bed bug bites and exposure to bed bug excrement can trigger systemic reactions such as asthma and anaphylaxis [25], [26]. Our survey of the whole insect microbiome uncovered at least 5 genera to which known or putative human pathogen species belong. For example, two OTUs of *Bacillus* were detected in three of our eight collection locations surveyed-other blood-feeding arthropods (mosquitos, horse and deer flies) have been documented to vector *B. anthracis*, the bacteria that causes anthrax [53]. We also found two OTUs in one collection location from the family that *Coxiella burnetti* (Q-fever) belongs to, along with two *Streptococcus* OTUs found in five populations and one *Staphylococcus* OTU found in four collection locations. While the use of short-read 16S rDNA sequences does not enable identification of the bacterial species and strains of interest to human health, the use of such technology across a broad area, followed by either 16S rDNA Sanger sequencing and/or high-depth metagenomic sequencing could identify human pathogens at the level of the species.

### Similar bacterial lineages across populations suggest host-microbe association

Our distance-based screen of 54,114 sequences from 31 individual *Cimex* libraries uncovered 359 OTUs-a number that is high relative to other surveys of arthropod bacterial communities (e.g., 139 OTUs found across species and wild populations of *Drosophila*, [9]; 74 OTUs uncovered across 11 *Drosophila* populations, [17]; both Sanger sequenced, long-read surveys). Our use of multiple individuals collected across geographically separate locations, the method of sample preparation, and 454 sequencing error are all factors that likely influenced this high number. To reduce sequencing error on our estimates, we followed the standard operating procedure from the Schloss lab (http://www.mothur.org/wiki/Main_Page), which included sequence quality checks and trimming of low-quality sequence and homopolymers, as well as a pre-clustering step that bins rare sequences that are within 2 bp of a more common OTU into the more common group. We also searched for and removed chimeric sequences within the database-such screens are reported to reduce the overall chimera rate to 1% [54]. Further, we utilized techniques that reduce PCR amplification errors such as using a high-fidelity Taq polymerase and the pooling of triplicate amplifications of each individual's DNA. With these checks in place, we still uncovered a large number of OTUs that were very low-copy (239 OTUs represented by 1 or 2 sequences). We removed these low-copy sequences from the overall dataset and performed each step of the presented analysis with OTUs represented by three or more sequences. This highly conservative treatment of the data uncovered 49 OTUs at the 3% level of divergence-many fewer than the 359 uncovered with the full dataset. However, all of our major conclusions were the same: collection locations exhibited variable diversity and we found a similar relative abundance of the 10 most abundant OTUs (data not shown). Although we cannot distinguish rare, environmental OTUs from 454 sequencing error with our full dataset, if we were to assume an error rate of 1% from non-identified chimeras, and another 18% error from 454 sequencing chemistry (as identified using the V4 region in [55]), we would reduce our number of OTUs by 68 to 291. Thus, it is reasonable to predict that many of the low-copy OTUs from across the eight collection locations are from environmental sources, such as bacteria picked up and transported on the surface of the individuals.

In comparison, the OTUs that we uncovered across all or most individuals and/or collection locations, and are represented by more than a few sequences, are likely to be either endosymbionts or consistently associated with the *C. lectularius* exoskeleton rather than strictly environmental. Ten such OTUs were uncovered in all locations, and 11 OTUs were found in 15 or more individuals, albeit most at very low frequency. Of note, *Methylobacterium* was found in 25 of the individual libraries and in all locations-this group of bacteria is often associated with plants. One species in particular, *M. mesophilicum*, is harbored by glassy-winged sharpshooters [56] and has been suggested as a sharpshooter biocontrol agent to control associated vector-borne plant diseases. An OTU classified as belonging to the *Sphingomonas* genus, having a widespread ecological distribution within water, soil and in association with plants [57], was uncovered in 22 individual bed bug libraries. Finally, in 21 libraries, we detected the presence of *Propionibacterium*, a genus of bacteria to which *P. acnes* belongs-these bacteria, which cause acne in humans, are often found on skin and infecting human blood [58], [59].

### Variation in the structure, diversity and evenness of the *Cimex* microbiome

We uncovered evidence of variation in diversity and evenness among the *Cimex* microbial collection locations. Whereas most of the Inverse Simpson values range from 1.8–2.1 among locations, locations 5 and 8 show comparatively lower values, from ∼1.3 to 1.4. These results are likely influenced by the relative abundances of the most abundant OTUs-*Wolbachia* dominated these two locations, whereas the other locations exhibited less extreme relative abundances of *Wolbachia* versus the unnamed γ-proteobacterium. Further, locations 5 and 8 did not exhibit wide differences in the abundance of the low-copy OTUs that were similarly uncovered in most locations, suggesting that the significant variation among locations in diversity and evenness are driven largely by the differences in abundance of *Wolbachia* and the unnamed γ-proteobacterium. A survey of the geographical distribution of the microbial community from natural *Drosophila melanogaster* populations has also uncovered population variation in community composition and taxon richness [17], and further, there is evidence that the major taxa of the lone star tick microbiome show heterogeneity among populations [10]. This survey of the *Cimex* microbiome is relatively unique compared to previous assessments [33] in that between 2 and 5 individuals were assessed separately per collection location. The use of multiple sequenced microbiomes per population thus allows us to definitely test for the presence of location differentiation among wild-caught *C. lectularius*, which has implications for understanding endosymbiont patterns of co-occurrence and for generating expectations regarding the frequency of potential human pathogenic bacteria.

The significant community structure among *C. lectularius* microbiomes from different collection locations that we uncovered in this survey may be due, in part, to the maternally inherited portion of the microbiome. Recent work shows that the population genetic structure of *C. lectularius* is high across the eastern US, with little variation within populations [60]. This pattern suggests multiple introductions of *C. lectularius* into the United States-and hence high diversity among populations-followed by subsequent inbreeding within populations due to the establishment of the population by a single mated female [60]. Our results of significant structure within the microbiome of *C. lectularius* among collection locations preliminarily supports this pattern since the dominant members of the microbiome are known to be maternally transmitted, and thus, the establishment of a new population *via* a single female would establish a maternally-inherited microbiome. That we uncovered community structure in the microbiomes between locations is even more striking in light of our experimental decision to assess the whole-insect microbiome, *i.e.*, environmental and endosymbiont bacteria together. However, this finding may also reflect differences among households in the bacteria present in the environment, and our data thus far cannot disentangle the relative influence of the environmentally-derived bacteria from the internal community on the overall microbiome structure.

## Conclusions

The data presented here provide a novel view of the microbial constituents of bed bugs across collection locations. That the relative abundances of *Wolbachia* and the γ-proteobacterium appeared to vary across locations suggest a range of interesting questions, such as: Might the two dominant members of the bed bug microbiome competitively exclude one another, or, does host fitness depend on the presence of both types in an additive fashion? Are the patterns of community structure and relative abundance of the two dominant groups from naturally collected locations strictly due to inheritance from a founding maternal individual, or might there be unexamined environmental influences? How might these patterns of community structure impact potential attempts to eradicate or control bed bug infestations? Further assessments of the core microbiome across multiple established bed bug populations will have broad implications for our understanding of bed bug physiology, human health, and bed bug biocontrol.

## Methods

### Sample collection and DNA isolation

*C. lectularius* samples from distinct collection locations were obtained from residences in the greater Cincinnati (OH, USA) area by Scherzinger Pest Control and generously donated to the Baucom lab for this project (see Fig 1 for locations). Technicians recorded the perceived severity of each infestation, which ranged from 5 (most severe) to 1 (least severe). Most donated populations were scored ‘5,’ with the exception of a single population that was scored as a ‘3’ (see Table S1 for specific information). The sizes of populations from collection locations were not otherwise characterized. In accordance with residential contracts, the pest company did not divulge the exact location of each residence; however, a general address was supplied for our records. Other identifying information about each residence, such as whether it was from an apartment building or single-family residence was not provided. Each *C. lectularius* population was collected from within a single residence. Between 10–50 individuals per collection location were randomly collected from the residence and placed in sterile 50 mL falcon tubes. Once collected, each sample of insects was starved for ∼7 days before being snap frozen in liquid nitrogen and stored at −20 °C until DNA isolation. Five individuals from each of the eight collection locations were randomly selected and weighed in sterile conditions. DNA was isolated from each individual using the Powersoil DNA Isolation Kit (MO Bio, Carlsbad, CA, USA) and tested for purity on a NanoDrop 2000 Spectrophotometer (ThermoScientific, Waltham, MA, USA). Across collection locations, average DNA yield was 3.97 (±0.62 SE) ng/uL with an average 260/280 score of 2.30 (±0.11 SE). Individuals were not surface sterilized prior to DNA isolation since the whole insect microbial community, and the community the insects potentially transport on their exteriors and/or in their feces were of interest.

### 16S Library and Sequencing

16S rDNA genes were PCR amplified in triplicate using the bacterial universal primers 530F (5′-GTGCCAGCMGCNGCGG-3′) and 1100R (5′-GGGTTNCGNTCGTTG-3′) which amplified∼600 bp corresponding to the V4–V6 hypervariable region of *Escherichia coli* [61]. DNA from each individual was amplified by PCR in order to create amplicon libraries for pyrosequencing using Titanium GS FLX chemistry on a 454 sequencing machine. Primers thus included the Titanium Fusion A or B primers and a 15 bp error-correcting mid-tag optimized for individual amplicon library identification from a single pyrosequencing run (Roche, Penzberg, DE); the general sequence of each primer was LinkerA-midtag-530F and LinkerB-1100R (see Table S2 for specific primers utilized in this study [62]). The HotStar HiFidelity Polymerase Kit (Qiagen, Valencia, CA, USA) was used for PCR under the following conditions: 95 °C for 5 min followed by 40 cycles of 94 °C for 15 s; 60 °C for 1 min; 72 °C for 1 min; and a final elongation of 72 °C for 10 min. Each sample was amplified in triplicate, pooled and cleaned using the QIAquick PCR Purification Kit (Qiagen, Valencia, CA, USA) followed by normalization of each library to 25 ng using the SequalPrep Normalization Plate Kit (Invitrogen, Carlsbad, CA, USA). After amplification, the PCR products were run on a 1% agarose gel, and samples that did not amplify well in triplicate were not included in subsequent steps. This reduced the total number of libraries to 31 (see STable1 for the number of libraries per collection location). Once normalized, libraries were pooled and sent to the Purdue Genomics Lab for pyrosequencing according to standard protocols (Roche, Penzberg, Germany).

### Sequence analysis

Sequence quality control was performed using mothur [36] in the following manner: first, the trim.seqs command was used to trim the sequence when the average quality score over a 50 bp window dropped below 35, and sequences with ambiguous bases (N), homopolymers (>8 bases), or sequences that were <200 bp were removed. The pdiffs = 2 and bdiffs = 1 options of this command were utilized in order to allow a maximum of 2 and 1 nucleotide differences in the primer and barcode sequences, respectively. Sequences were aligned to the SILVA-compatible alignment database using align.seqs, and then trimmed to a common region. To further reduce the influence of pyrosequencing error on downstream analysis, sequences were clustered using the pre.cluster command, and those that were within 2 bp of similarity to a more abundant sequence were merged with the more abundant type. Next, potential chimeric sequences were removed from the full dataset by utilizing the SILVA gold database as the reference in the chimera.uchime command. Finally, the classify.seqs and remove.lineage commands were utilized to identify and remove potential mitochondrial and chloroplast contaminants.

Bacterial taxonomy was assigned to each sequence in the improved dataset using the classify.seqs command, which implements the Naïve Bayesian classifier of RDP, as implemented in mothur [36]. Following taxonomic assignment, sequences were assigned to OTUs using the cluster command, following which the consensus taxonomy for each OTU at the 3% level of divergence was determined using the classify.otu command [36]. The abundance of each OTU_0.03_ within each individual and collection location was determined using the shared.otu command. Broad patterns of the presence or absence of specific OTUs across all *Cimex* populations were considered utilizing the improved, full dataset. To compare estimates of diversity and community structure among collection locations, we first normalized the data such that 1000 sequences were randomly subsampled from each individual. Individuals with <1000 sequences following quality control were thus excluded from further analyses (Table S1).

Commands within the program mothur were used to produce rarefaction curves and assess both alpha- and beta-diversity of the communities. The coverage, number of observed OTUs_0.03_, the Shannon evenness and the Inverse Simpson statistics were calculated for each individual, and an ANOVA using Type I SS was performed in the R statistical programming language (http://www.R-project.org) to determine if collection locations varied in these estimates of microbial community diversity, after log_10_ transforming dependent variables to improve the normality of residuals. To describe the community dissimilarity, (and conversely, similarity in community membership and structure across locations) the Yue and Clayton theta statistic was estimated via the dist.shared command. The resulting distance matrix was visualized in a principle components ordination plot using the first two axes, and then a rotation of the 3D matrices was performed using the ggobi package in R [63].

We next performed an analysis of molecular variance using the amova command of mothur to determine if there was significant structure among collection locations, followed by the metastats command to identify OTUs_0.03_ that were differentially represented in the separate *Cimex* populations. The presence of a core microbiome was assessed by first using the make.shared command to determine which OTUs were shared across all individuals and by then visualizing the abundance of OTUs using the heatmap.2 command of R. Sequences from this study are deposited in Dryad (doi:10.5061/dryad.rd240) and are also available from RS Baucom upon request.

## Supporting Information

Figure S1 **Rarefaction of the Inverse Simpson index for each** ***Cimex*** **library.**
(TIF)

Table S1 **General location of populations and the number of individuals used in the taxonomic, diversity and abundance screens.**
(DOCX)

Table S2 **The abundance of all OTUs within the Cimex populations.**
(XLSX)

Table S3 **Analysis of variance for assessing the effect of location on variation in estimates of A.** Inverse Simpson and B. Shannon Evenness indices.
(DOCX)

Table S4 **The coverage, number of observed OTUs, and the Inverse Simpson, Shannon Evenness, and the non-parametric Shannon diversity index.**
(XLSX)

Movie S1 **A rotating PCoA plot of community similarity (Yue and Clayton theta) among individual bed bug microbiomes.** The rotation of the first 2 axes from the principle components ordination plot was performed by ggobi package in R.
(MOV)
