# Supplementary figures and images for: A 454 Survey Reveals the Community Composition and Core Microbiome of the Common Bed Bug (Cimex lectularius) across an Urban Landscape
Source: PLoS One. 2013 Apr 9;8(4):e61465. doi: 10.1371/journal.pone.0061465 (PMC3621965; doi:10.1371/journal.pone.0061465)

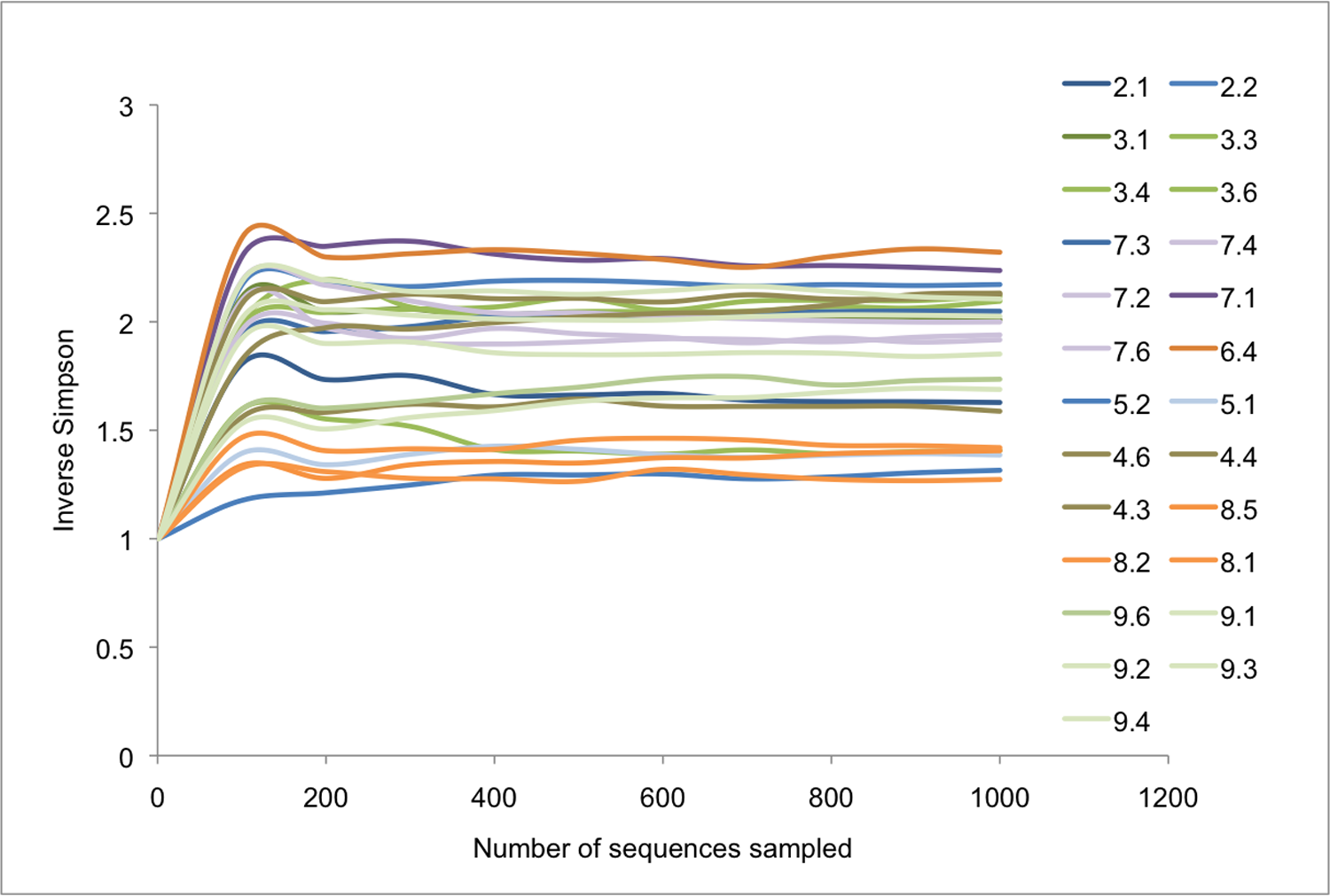

Supplement: Figure S1 — Rarefaction of the Inverse Simpson index for each Cimex library. (TIF) [file pone.0061465.s001.tif]
