# Supplementary material for: A 454 Survey Reveals the Community Composition and Core Microbiome of the Common Bed Bug (Cimex lectularius) across an Urban Landscape
Source: PLoS One. 2013 Apr 9;8(4):e61465. doi: 10.1371/journal.pone.0061465 (PMC3621965; doi:10.1371/journal.pone.0061465)
Supplement: Table S1 — General location of populations and the number of individuals used in the taxonomic, diversity and abundance screens. (DOCX) [file pone.0061465.s002.docx]

|  |  |  |  |  |
| --- | --- | --- | --- | --- |
| Location name | Severity of infestation, as noted by technician | General Location | Number of individuals assayed per location | Number of individuals with 1000 sequences |
| loc2 | 5 | Loveland | 3 | 2 |
| loc3 | 5 | Damont | 5 | 4 |
| loc4 | 5 | Galbraith | 4 | 3 |
| loc5 | 5 | Drummond | 3 | 2 |
| loc6 | 3 | Price Hill | 2 | 1 |
| loc7 | 5 | Mt. Vernon | 5 | 5 |
| loc8 | 5 | Clifton | 4 | 3 |
| loc9 | 5 | Oak Dr. | 5 | 5 |
|  |  |  |  |  |

STable1. General location of populations and the number of individuals used in the taxonomic, diversity and abundance screens.
