# Supplementary material for: A 454 Survey Reveals the Community Composition and Core Microbiome of the Common Bed Bug (Cimex lectularius) across an Urban Landscape
Source: PLoS One. 2013 Apr 9;8(4):e61465. doi: 10.1371/journal.pone.0061465 (PMC3621965; doi:10.1371/journal.pone.0061465)
Supplement: Table S3 — Analysis of variance for assessing the effect of location on variation in estimates of A. Inverse Simpson and B. Shannon Evenness indices. (DOCX) [file pone.0061465.s004.docx]

|  |  |  |  |  |  |
| --- | --- | --- | --- | --- | --- |
| A | Source of variation | df | SS | F | P-value |
|  | Location | 6 | 0.123 | 4.47 | 0.009 |
|  | Residual | 15 | 0.069 |  |  |
|  |  |  |  |  |  |
| B | Source of variation | df | SS | F | P-value |
|  | Location | 6 | 0.086 | 4.84 | 0.005 |
|  | Residual | 16 | 0.047 |  |  |
|  |  |  |  |  |  |

STable 3. Analysis of variance for assessing the effect of location on variation in estimates of A. Inverse Simpson and B. Shannon Evenness indices.
